# Sex-dependent Differences in the Bioenergetics of Liver and Muscle Mitochondria from Mice Containing a Deletion for *glutaredoxin-2*

**DOI:** 10.3390/antiox8080245

**Published:** 2019-07-26

**Authors:** Sarah Mallay, Robert Gill, Adrian Young, Ryan J. Mailloux

**Affiliations:** Department of Biochemistry, Memorial University of Newfoundland, St. John’s, NL A1B 3X7, Canada

**Keywords:** glutaredoxin-2, sex dimorphism, mitochondrial bioenergetics, reactive oxygen species, glutathionylation

## Abstract

Our group recently published a study demonstrating that deleting the gene encoding the matrix thiol oxidoreductase, glutaredoxin-2 (GRX2), alters the bioenergetics of mitochondria isolated from male C57BL/6N mice. Here, we conducted a similar study, examining H_2_O_2_ production and respiration in mitochondria isolated from female mice heterozygous (GRX2+/−) or homozygous (GRX2−/−) for glutaredoxin-2. First, we observed that deleting the *Grx2* gene does not alter the rate of H_2_O_2_ production in liver and muscle mitochondria oxidizing pyruvate, α-ketoglutarate, or succinate. Examination of the rates of H_2_O_2_ release from liver mitochondria isolated from male and female mice revealed that (1) sex has an impact on the rate of ROS production by liver and muscle mitochondria and (2) loss of GRX2 only altered ROS release in mitochondria collected from male mice. Assessment of the bioenergetics of these mitochondria revealed that loss of GRX2 increased proton leak-dependent and phosphorylating respiration in liver mitochondria isolated from female mice but did not alter rates of respiration in liver mitochondria from male mice. Furthermore, we found that deleting the *Grx2* gene did not alter rates of respiration in muscle mitochondria collected from female mice. This contrasts with male mice where loss of GRX2 substantially augmented proton leaks and ADP-stimulated respiration. Our findings indicate that some fundamental sexual dimorphisms exist between GRX2-deficient male and female rodents.

## 1. Introduction

Protein S-glutathionylation is a ubiquitous and reversible redox-sensitive protein modification that has emerged as an important signal for the control of cell functions. Mitochondria are highly susceptible to protein S-glutathionylation due to the physical properties of the matrix. First, the matrix is slightly basic, promoting thiol ionization and the formation of nucleophilic thiolate anions, factors that promote S-glutathionylation [1]. Mitochondria are also enriched in GSH (~ 2 mM) and are an important source of cellular reactive oxygen species (ROS), a driving force behind S-glutathionylation reactions [2]. Glutaredoxin-2 (GRX2), a homolog of cytosolic GRX1, catalyzes the reversible S-glutathionylation of proteins in the matrix of mitochondria. Complex I in bovine heart mitochondria was the first GRX2 target that was identified [3]. The same study also demonstrated that GRX2 reversibly modifies Complex I in response to changes in the redox state of the GSH pool [3]. GRX2 also targets uncoupling protein-3 (UCP3) for S-glutathionylation in muscle and α-ketoglutarate dehydrogenase (KGDH) and pyruvate dehydrogenase (PDH) in liver mitochondria [4,5,6,7]. In addition, mitochondria from liver, cardiac, and muscle tissue contain a number of S-glutathionylation targets, which includes proteins involved in oxidative phosphorylation, fuel combustion, apoptosis, cell signaling, solute transport, and mitochondrial fission/fusion (reviewed in [2,8]).

Mitochondria are the most important source of ROS in mammalian cells. The proximal ROS formed by mitochondria are superoxide (O_2_●^−^) and hydrogen peroxide (H_2_O_2_), which are generated by the same fuel-oxidizing and electron-transferring enzymes that make ATP [9,10]. Hydrogen peroxide serves as a second messenger required for mitochondria-to-cell communication [11]. Protein S-glutathionylation is one of several mechanisms that mitochondria use to control H_2_O_2_ availability for cell signaling. Evidence collected so far indicates that S-glutathionylation serves as a negative feedback loop that shuts down ROS production in response to oxidation of mitochondrial redox buffering networks. For example, oxidation of glutathione pools using diamide or disulfiram results in the S-glutathionylation of Complex I and inhibition of H_2_O_2_ production in skeletal muscle [12]. Additionally, reversible S-glutathionylation of Complex I by GRX2 in response to changes in the redox buffering capacity also controls H_2_O_2_ production. Oxidation of glutathione pools results in the GRX2-mediated S-glutathionylation of Complex I inhibiting ROS production [3,13]. Reduction of redox buffering networks has the opposite effect, deglutathionylating Complex I and restoring its ROS generating activity [3,13]. Recent work has also found that oxidation of glutathione pools results in the S-glutathionylation of pyruvate dehydrogenase (PDH) and α-ketoglutarate dehydrogenase (KGDH), abolishing H_2_O_2_ release in liver mitochondria [6]. Purified GRX2 deglutathionylates both enzyme complexes, restoring their activity and ROS generating capacity [6,14]. In addition, S-glutathionylation can modulate ROS production indirectly by deactivating solute uptake by mitochondria. This can be achieved through the S-glutathionylation of pyruvate carrier protein, which curtails H_2_O_2_ production by inhibiting pyruvate uptake [12]. GRX2 and S-glutathionylation also regulate mitochondrial ROS production through the reversible S-glutathionylation of UCP3 [15,16].

Our group recently published a study demonstrating that GRX2 is required to regulate the bioenergetics of mitochondria isolated from male mice [17]. Deletion of the *Grx2* gene resulted in increased PDH and KGDH S-glutathionylation, which diminished ROS production by both enzyme complexes [17]. It was also found that the loss of GRX2 augmented H_2_O_2_ production during succinate oxidation [17]. Furthermore, in a separate study, we observed that S-glutathionylation is critical for regulating the bioenergetics of skeletal muscle mitochondria from male rodents [12]. Armed with this information, we decided to compare the bioenergetics of liver and muscle mitochondria isolated from female and male mice containing a deletion for the *Grx2* gene. Here, we present findings demonstrating that deleting the *Grx2* gene does not alter the rate of H_2_O_2_ production in liver and skeletal muscle mitochondria oxidizing pyruvate, α-ketoglutarate, or succinate. This is in contrast with mitochondria from male mice where 1) ROS production was significantly lower in liver samples from GRX2+/− and GRX2−/− mice and 2) loss of GRX2 increased ROS production when succinate was the substrate. Furthermore, analysis of the different states of respiration revealed that liver and muscle mitochondria display fundamental variances in fuel combustion and O_2_ consumption.

## 2. Experimental

### 2.1. Chemicals

Amplex Ultra Red (AUR) reagent was purchased from Invitrogen (Waltham, MA USA). Pyruvate, α-ketoglutarate, malate, succinate, HEPES, sucrose, mannitol, ethylene glycol-bis(β-aminoethyl ether)-N,N,N′,N′-tetraacetic acid (EGTA), fatty acid free (defatted) bovine serum albumin, hydrogen peroxide (30% solution) Bradford reagent, horse radish peroxidase (HRP), superoxide dismutase (SOD), KH_2_PO_4_, and MgCl_2_ were purchased from Sigma-Aldrich (St. Louis, MI, USA).

### 2.2. Animals and Genotyping

The GRX2 constitutive knockout model was originally generated by Dr. Ho’s laboratory by deleting exon 2 in the *Grx2* gene [18]. This results in the formation of a truncated *Grx2* mRNA sequence comprised of exons 1, 3, and 4 [17,18]. The translation product of the truncated *Grx2* mRNA sequence is then subjected to rapid degradation, which is evidenced by the complete absence of GRX2 in mitochondria [13,17]. C57BL/6N mice heterozygous for the *Grx2* gene were a gift from Dr. Mary-Ellen Harper (University of Ottawa). Wild-type (WT), *Grx2* heterozygous (GRX2+/−), and *Grx2* homozygous (GRX2−/−) littermates were produced by breeding age-matched male and female mice on a C57BL/6N background heterozygous for the *Grx2* gene. Animals were fed a standard chow diet (Teklad Global 18% protein rodent diet) and water ad libitum and housed in the Memorial University of Newfoundland animal care unit (~23 °C, 12 h dark/12 h light cycle with lights on at 7:00). New litters were weaned and males and females were ear notched for genotyping, as described previously [17]. Mice at ~10 weeks of age were weighed and euthanized by cervical dislocation under heavy isoflurane anesthesia to dissect liver and muscle tissue. Animal protocols and experiments were certified by Memorial University of Newfoundland’s Institutional Animal Care and Use Committee and conducted according to institutional and federal guidelines (Animal Care Ethics Protocol: 16-01-RM).

DNA extraction was performed using a REDExtract-N-Amp Tissue PCR Kit (Sigma-Aldrich, St. Louis, MI, USA) according to the manufacturer’s instructions. Primer sequences for amplification were obtained from Integrated DNA Technologies for the *Grx2* gene [5’-GAC CTA GCC TAC CAG ACT TGG CTG AAA TTT ATT C-3’(forward), 5’-CAT AGA CAC TCT TCA CTT TCA AGC CCA CCC TC-3’ (reverse), 5’-CCT ACA TTT TGA ATG GAA GGA TTG GAG CTA CGG G-3’ (neo)]. DNA sequences were amplified using an Eppendorf ep gradient Mastercycler PCR system. PCR samples were loaded into a 1.5% (*w/v*) agarose gel containing SYBR Safe DNA Gel Stain (1/1000, Thermo Fisher Scientific) along with a 100 base pair (bp) DNA Ladder (Thermo Fisher Scientific). The gel was then electrophoresed for 40 min at 90 V and imaged on an Alpha Innotech ChemiImager Ready System. Wild-type mice produced a band corresponding to a fragment size of 729 bp in length, while *Grx2-/-* mice produced a nucleotide fragment of 510 bp and the *Grx2+/-* mice contained both fragments (510bp and 710bp) [17].

### 2.3. Mitochondrial Preparation

Liver and muscle mitochondria were prepared as described in [12,17]. All steps were performed on ice or at 4 °C. Liver tissue was removed and placed in a buffer containing 220 mM mannitol, 1 mM EGTA, 70 mM sucrose, and 20 mM HEPES (pH 7.4, MESH). Liver tissue was then cut into small pieces, rinsed with MESH to remove excess blood and fat, and then minced on a Teflon plate. Minced tissue was then homogenized in 25 mL of MESH supplemented with 0.5% *w/v* delipidated BSA (MESH-B). The homogenate was then centrifuged at 800× g for 9 min. The supernatant was collected and centrifuged at 12, 000× g for 9 min. The resulting mitochondrial pellet was then washed in MESH-B and centrifuged again at 12,000× g. Mitochondria were resuspended in 500 µL of MESH, giving a final concentration of ~16–18 mg/mL.

Dissected muscles from the fore and hind limbs and pectoral region were washed in basic medium (BM; 140 mM KCl, 20 mM HEPES, 5 mM MgCl_2_, 1 mM EGTA, pH adjusted to 7.0). Pooled muscles were cleaned of connective tissues, weighed, and then minced on a Teflon plate. Minced tissues were then placed in homogenizing medium (HM; BM + 1 mM ATP, 1% *w/v* defatted BSA, and 1 U of subtilisin A). Tissues were homogenized using a Glas-Col Variable Speed Tissue Homogenizer (Cole Parmer, Vernon Hills, Il, USA) and the homogenate was centrifuged at 800× g for 9 min. The supernatant was then collected and centrifuged at 12,000× g for 9 min to pellet mitochondria and myofibers. The pellet was resuspended in 1 mL BM and incubated on ice for 5 min and then, the suspension was diluted further by adding 25 mL of ice-cold BM. Samples were centrifuged again at 800× g to pellet repolymerized myofibers. The supernatant was carefully collected to avoid disturbing the pellet and centrifuged at 12,000× g. The resulting mitochondrial pellet was resuspended in 200 µL of BM, giving a final protein concentration equivalent to mitochondria of ~9–12 mg/mL. Mitochondrial protein concentrations were quantified using the Bradford Assay and defatted BSA as the standard.

### 2.4. Quantification of Hydrogen Peroxide Production

The rate of H_2_O_2_ release by mitochondria into the surrounding extramitochondrial environment was measured using the Amplex UltraRed (AUR) Assay as described in [19,20]. Liver mitochondria were diluted to a final concentration of 3 mg/mL in MESH. Muscle mitochondria, on the other hand, were diluted to 1.5 mg/mL in BM. Diluted samples were then stored on ice. Samples were then diluted further in MESH (liver) or BM (muscle) to 0.3 and 0.15 mg/mL, respectively, in the reaction chambers of a clear-bottom, black 96-well plate. Mitochondria were equilibrated in the buffer for a few minutes at 25 °C in the plate reader and then the reaction mixtures were supplemented with HRP (3 U/mL), SOD (25 U/mL), and AUR (10 µM). Reactions were commenced following the addition of pyruvate, α-ketoglutarate, or succinate to a final concentration of 10–10,000 µM. The rate of H_2_O_2_ production was measured using a SpectraMax plate reader (Molecular Devices, San Jose, CA, USA) and Softmax Pro software (version 5.4.6) set to an excitation: emission wavelengths of 565:600 nm. The rate of H_2_O_2_ production was calculated using AUR standard curves and values were corrected for background fluorescence.

### 2.5. Measurement of Mitochondrial Respiration: 

Mitochondrial bionenergetics were evaluated by measuring the different states of respiration using an Oxytherm Clark electrode system (PP Systems, Amesbury, MA, USA). Liver and muscle mitochondria were diluted to 0.5 mg/mL and 0.2 mg/mL, respectively, in the reaction chamber which contained either BM supplemented with 10 mM KH_2_PO_2_, 5 mM MgCl_2_, and 0.1% *w/v* delipidated BSA (muscle) or MESH-B containing 10 mM KH_2_PO_2_ and 5 mM MgCl_2_ for liver. Mitochondria were incubated in the chamber under state 1 conditions until a stable O_2_ baseline was reached and then pyruvate (10 mM) and malate (2 mM) or succinate (5 mM) were injected into the chamber to induce state 2 respiration. State 2 respiration was measured for ~4 min. State 3 respiration was then induced and measured by adding ADP (1 mM). Once the ADP was exhausted in the reaction chamber, which is denoted by a significant decrease in O_2_ consumption, state 4 respiration was assessed by adding oligomycin (4 μg/mL) to the reaction chamber. Antimycin A (4 μM) was then added to arrest the respiratory chain and measure background O_2_ consumption. The respiratory values for States 2–4 were corrected for background oxygen consumption (oxygen consumption minus onsumption recorded after antimycin A addition) and normalized to the protein concentration.

### 2.6. Data Analysis

All experiments were conducted 4 times and in duplicate or triplicate to account for experimental error. Raw data calculations were conducted using Microsoft Excel. Results generated using Excel were analyzed with GraphPad Prism 6 software (San Diego, CA, USA) using 1-way and 2-way ANOVA with a Tukey’s posthoc test or a paired two-tailed Student’s T-Test. * or #: *p* ≤ 0.05, ** or ##: *p* ≤ 0.01, *** or ###: *p* ≤ 0.001. 

## 3. Results

### 3.1. Loss of GRX2 does not Alter H_2_O_2_ Production in Liver Mitochondria

**Figure 1:**
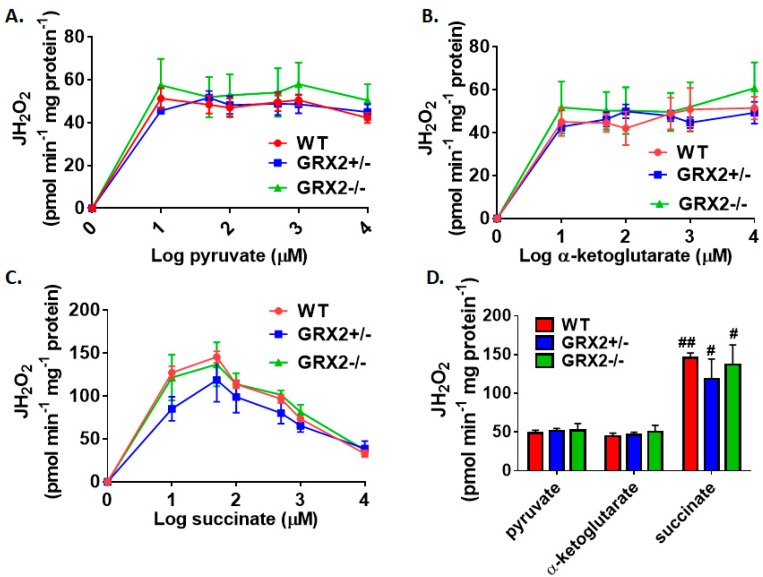
Deletion of the *Grx2* gene does not alter the rate of H_2_O_2_ production by liver mitochondria isolated from female mice. Rates of production by mitochondria energized with pyruvate (**A**), α-ketoglutarate (**B**), or succinate (**C**) at a concentration range of 10–10,000 µM. (**D**). Comparison of the rates of reactive oxygen species (ROS) production in mitochondria isolated from wild-type (WT), GRX2+/−, and GRX2−/− mice oxidizing 50 µM pyruvate, α-ketoglutarate, or succinate. *n* = 4, mean ± SEM (standard error of mean), 2-way ANOVA with a post-hoc Tukey’s test. #: *p* ≤ 0.05, ##: *p* ≤ 0.01.

Figure 1A–C demonstrate that the max rate for H_2_O_2_ production in liver mitochondria prepared from female mice is achieved at 10–50 µM for pyruvate, α-ketoglutarate, and succinate. No significant differences in H_2_O_2_ production between WT, GRX2+/−, and GRX2−/− liver mitochondria at all concentrations of pyruvate, α-ketoglutarate, or succinate used in these experiments were observed (Figure 1A–C). In addition, the rate of ROS generation by mitochondria starts to decay at higher succinate concentrations (Figure 1C). This would indicate that ROS production by Complex II is subjected to product inhibition, an observation that is consistent with a previously published study [21]. Next, we compared the rates of H_2_O_2_ production in liver mitochondria energized with 50 µM substrate. Upon energization, we were able to confirm that there was no difference in H_2_O_2_ production between the three genotypes metabolizing pyruvate, α-ketoglutarate, or succinate (Figure 1D). It was also found that succinate was a more potent inducer of ROS production, resulting in a ~3-fold increase in ROS production when compared to pyruvate (Figure 1D). These results establish that loss of GRX2 does not alter H_2_O_2_ release from liver mitochondria prepared from female C57BL/6N mice.

### 3.2. GRX2 Deficiency Alters ROS Release from Liver Mitochondria Prepared from Male Mice Only

**Figure 2:**
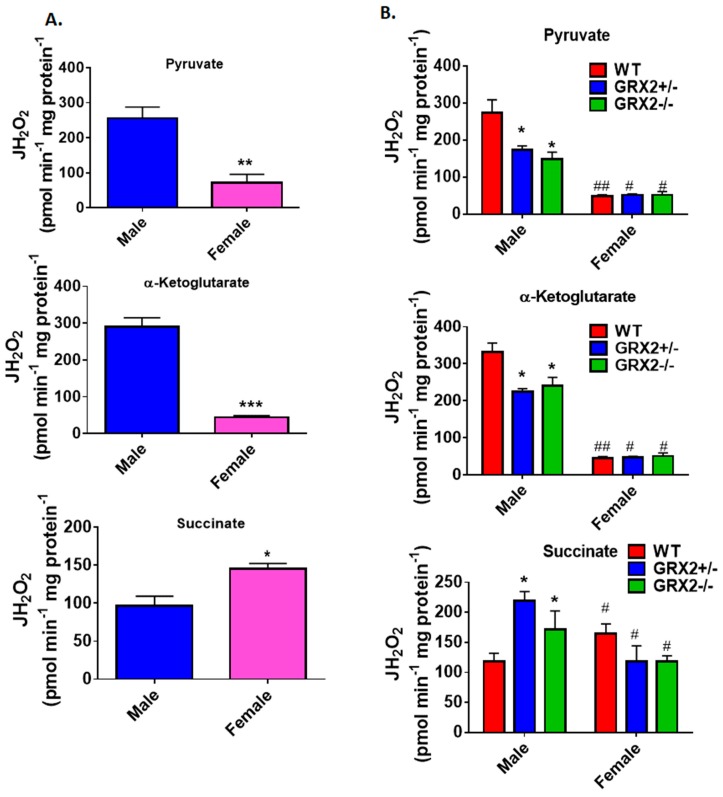
Examination of the sex differences associated with H_2_O_2_ production by liver mitochondria isolated from female and male littermates deficient for GRX2. (**A**). Comparison of the rate of H_2_O_2_ production by liver mitochondria isolated from male and female WT littermates and energized with 50 µM pyruvate, α-ketoglutarate, or succinate. *n* = 4, mean ± SEM, two-tailed Student’s *T*-Test. (**B**). Impact of GRX2 deficiency on rates of ROS production by liver mitochondria from male and female mice. *n* = 4, mean ± SEM, 2-way ANOVA with a post-hoc Tukey’s test, * represents a genotype comparison within a group and # represents a sex comparison. * or #: *p* ≤ 0.05, ** or ##: *p* ≤ 0.01, *** or ###: *p* ≤ 0.001.

A previously published study by our group showed that deleting the *Grx2* gene had opposite effects on ROS production from male liver mitochondria energized with pyruvate, α-ketoglutarate, or succinate [17]. Loss of GRX2 significantly diminished ROS production from mitochondria oxidizing pyruvate or α-ketoglutarate whereas it had the opposite effect on succinate metabolism [17]. Based on our findings above, we decided to compare the ROS production profiles of male and female littermates oxidizing these three substrates to ascertain if sex affected H_2_O_2_ production. Figure 2A shows that mouse sex has a significant impact on the rate of H_2_O_2_ production when pyruvate, α-ketoglutarate, or succinate are fueling ROS genesis. Liver mitochondria prepared from male WT mice generated ~3.5-fold and ~5-fold more ROS than their female counterparts when pyruvate or α-ketoglutarate, respectively, were serving as substrates (Figure 2A). Intriguingly, the opposite effect was observed in female liver mitochondria energized with succinate; a small but significant increase in H_2_O_2_ formation was observed when compared to male WT littermates (Figure 2A).

Next, the impact of mouse sex on ROS production in liver mitochondria prepared from GRX2+/− and GRX2−/− mice was examined. First, it was found that liver mitochondria from female mice displayed significantly lower rates for ROS production when oxidizing pyruvate or α-ketoglutarate, regardless of genotype (Figure 2B). Secondly, loss of GRX2 impacted ROS production in liver mitochondria from male mice only. Indeed, liver mitochondria from male mice heterozygous and homozygous for the *Grx2* gene generated significantly less H_2_O_2_ when compared to male WT littermates when pyruvate and α-ketoglutarate served as the substrate (Figure 2B). In contrast, no differences were observed in liver mitochondria prepared from GRX2-deficient and WT female mice. We also examined the impact of sex and genotype on ROS production in the presence of succinate. First, liver mitochondria from WT female mice generated significantly more ROS than their male WT counterparts (Figure 2B). The second critical observation made was that there was a genotype effect in male mice only. Indeed, liver mitochondria from male GRX2+/− and GRX2−/− mice displayed a significant increase in the rate of H_2_O_2_ production (Figure 2B). However, no such effect was observed in liver mitochondria isolated from female littermates (Figure 2B). These revelations indicate that there is a sexual dimorphism associated with the GRX2-mediated regulation of ROS production in male and female liver mitochondria.

### 3.3. Impact of Deleting the Grx2 Gene on Liver Mitochondria Bioenergetics

**Figure 3:**
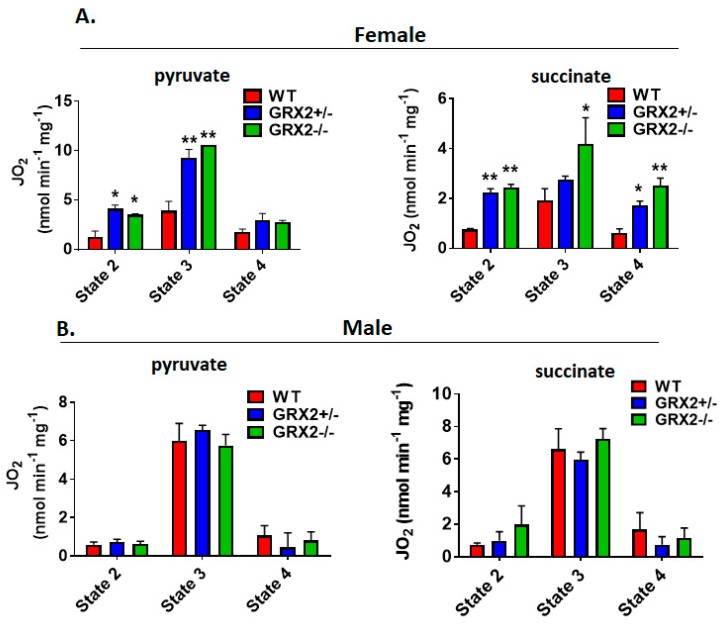
Polarographic measurement of the rate of O_2_ consumption by liver mitochondria isolated from female and male GRX2-deficient mice and WT littermates. (**A**). Assessment of the three respiratory states of mitochondria from female mice oxidizing pyruvate (10 mM) and malate (2 mM) or succinate (5 mM). (**B**). Assessment of the three respiratory states of mitochondria from male mice oxidizing pyruvate (10 mM) and malate (2 mM) or succinate (5 mM). *n* = 4, mean ± SEM, 1-way ANOVA with a post-hoc Tukey’s test. *: *p* ≤ 0.05, **: *p* ≤ 0.01.

Next, we measured the different states of respiration since the same fuel-oxidizing and electron-transferring enzymes required to make ATP also generate ROS. To do so, we first energized mitochondria with pyruvate and malate or succinate to induce State 2 respiration (proton leaks). State 2 respiration when pyruvate and malate served as the substrate was significantly higher in female liver mitochondria prepared from GRX2+/− and GRX2−/− mice when compared to samples from WT littermates (Figure 3A). Similar observations were made with liver mitochondria energized with succinate (Figure 3A). Next, liver mitochondria were supplemented with ADP to stimulate State 3 respiration. GRX2+/− and GRX2−/− liver mitochondria energized with pyruvate and malate displayed significantly higher rates of O_2_ consumption in the presence of ADP (Figure 3A). Succinate induced a similar effect (Figure 3A). After measuring State 3 respiration, oligomycin was added to respiratory chambers to probe the rate of proton-leak-dependent respiration when ATP synthase is deactivated (State 4). No significant differences in State 4 respiration were observed during pyruvate and malate oxidation, although a trend for an increase was observed in GRX2+/− and GRX2−/− liver mitochondria (Figure 3A). However, State 4 respiration was ~2-fold higher in GRX2-deficient mitochondria metabolizing succinate (Figure 3A).

Conducting the same measurements using liver mitochondria enriched from male littermates revealed completely different trends. Indeed, deletion of the *Grx2* gene did not alter the rate of O_2_ consumption under State 2, 3 or 4 respiratory conditions, regardless of substrate (Figure 3B). Furthermore, although liver mitochondria from male and female WT littermates exhibited similar rates of respiration under State 2–4 respiratory conditions, the rate of ADP-stimulated respiration in samples collected from GRX2-deficient female mice was almost 2-fold higher when compared to their male counterparts (Figure 3A,B). Overall, these findings indicate that a sex difference exists for the GRX2-mediated modulation of bioenergetics in liver tissue, where glutathionylation is required to control ATP output in female but not male hepatocytes.

### 3.4. Effect of GRX2 Deficiency on the Bioenergetics of Skeletal Muscle Mitochondria

**Figure 4:**
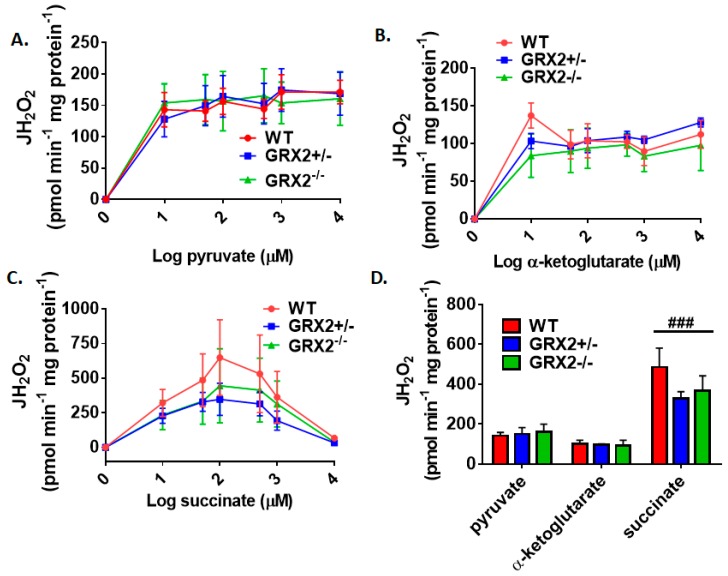
Deletion of the *Grx2* gene does not alter the rate of H_2_O_2_ production by muscle mitochondria isolated from female mice. Rates of production by mitochondria energized with pyruvate (**A**), α-ketoglutarate (**B**), or succinate (**C**) at a concentration range of 10–10,000 µM. (**D**). Comparison of the rates of ROS production in mitochondria isolated from WT, GRX2+/−, and GRX2−/− mice oxidizing 50 µM pyruvate, α-ketoglutarate, or succinate. *n* = 4, mean ± SEM, 2-way ANOVA with a post-hoc Tukey’s test. ###: *p* ≤ 0.001

Several studies have focused on the role of GRX2 and S-glutathionylation in regulating the bioenergetics of muscle mitochondria from male rodents [7,12,15]. Muscle mitochondria isolated from male mice homozygous for *Grx2* produce more ROS, an effect that is related to increased fuel combustion [15]. Additionally, protein S-glutathionylation of Complex I blunts ROS production and phosphorylating and proton leak-dependent respiration in muscle mitochondria prepared from male mice [12]. With this in mind, we next examined if sex affected the bioenergetics of skeletal muscle mitochondria isolated from GRX2-deficient female mice. First, it was observed that the maximum rate for H_2_O_2_ production was obtained at ~50 µM pyruvate and α-ketoglutarate, and succinate (Figure 4A–C). One exception was WT muscle mitochondria oxidizing succinate, which appeared to obtain its max rate for ROS production at ~250 µM (Figure 4C). We also observed product inhibition for ROS production at higher succinate concentrations for all genotypes (Figure 4C). The second observation made in Figure 4A–C was that there was no genotype effect. Indeed, rates for H_2_O_2_ production did not differ significantly between WT, GRX2+/−, and GRX2−/− muscle mitochondria oxidizing pyruvate, α-ketoglutarate, or succinate at different concentrations.

### 3.5. Sex Differences in ROS Production by Muscle Mitochondria Isolated from GRX2-Deficient Mice

**Figure 5:**
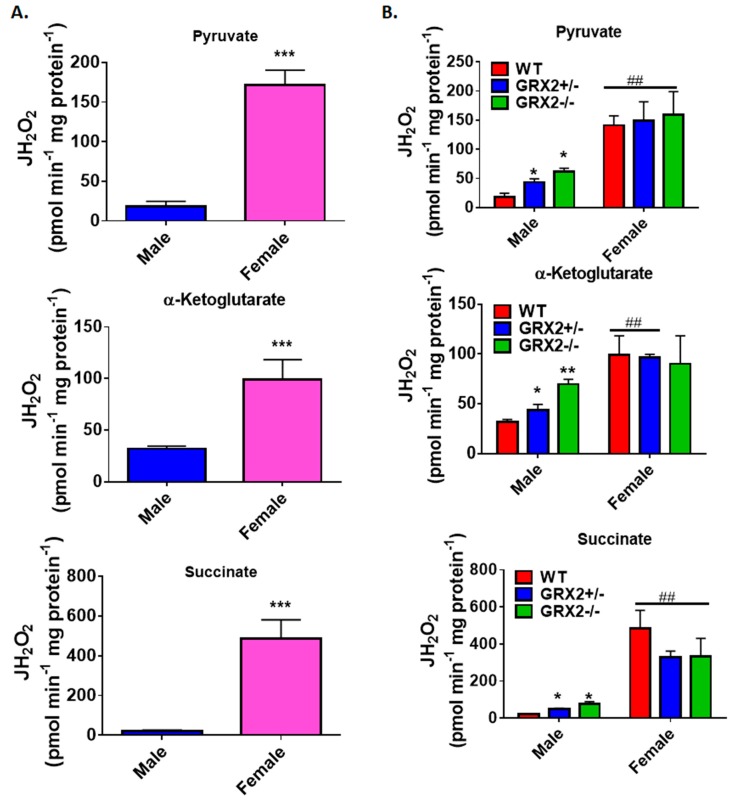
Examination of the sex differences associated with H_2_O_2_ production by muscle mitochondria isolated from female and male littermates deficient for GRX2. (**A**). Comparison of the rate of H_2_O_2_ production by muscle mitochondria isolated from male and female WT littermates and energized with 50 µM pyruvate, α-ketoglutarate, or succinate. *n* = 4, mean ± SEM, two-tailed Student’s *T*-Test. (**B**) Impact of GRX2 deficiency on rates of ROS production by muscle mitochondria from male and female mice. *n* = 4, mean ± SEM, 2-way ANOVA with a post-hoc Tukey’s test. *: *p* ≤ 0.05, ** or ##: *p* ≤ 0.01, ***: *p* ≤ 0.001

Next, we determined if a sex dimorphism exists for ROS production by comparing rates of H_2_O_2_ genesis by muscle mitochondria isolated from male and female mice. First, we found that female muscle mitochondria isolated from WT mice generate more H_2_O_2_ than samples collected from their male counterparts (Figure 5A). Indeed, muscle mitochondria from WT female mice generated upwards of ~2.5–3-fold more H_2_O_2_ when oxidizing Krebs-cycle-linked substrates, pyruvate or α-ketoglutarate (Figure 5A). In addition, similar results were collected with mitochondria energized with succinate, where the rate of ROS production was ~5-fold higher in comparison to mitochondria isolated from WT male mice (Figure 5A).

Following this, we investigated the impact of deleting the *Grx2* gene on ROS production by muscle mitochondria isolated from male and female mice. Similar to Figure 5A, we observed that sex had a profound impact on ROS production, with mitochondria from female mice generating more H_2_O_2_ than samples collected from male mice (Figure 5B). Additionally, this trend remained the same regardless of whether or not GRX2 was present. Indeed, mitochondria from female mice heterozygous or homozygous for GRX2 displayed no change in ROS production, regardless of substrate, when compared to female WT littermates (Figure 5B). However, a genotype effect was observed for male mice deficient in GRX2. Muscle mitochondria oxidizing pyruvate, α-ketoglutarate, or succinate from GRX2+/− male mice displayed a significant increase in ROS production when compared to WT littermates (Figure 5B). However, these rates of production were still much lower when compared to H_2_O_2_ genesis by mitochondria from female mice. Mitochondria from male mice homozygous for GRX2 displayed a further increase in ROS production when pyruvate, α-ketoglutarate, or succinate served as substrates (Figure 5B). In addition, rates of production in GRX2−/− muscle mitochondria from male mice when α-ketoglutarate served as the substrate were almost as high as the rate of genesis by mitochondria from GRX2-deficient females (Figure 5B).

### 3.6. Sex Differences in the Bioenergetics of Muscle Mitochondria from GRX2-Deficient Mice 

**Figure 6:**
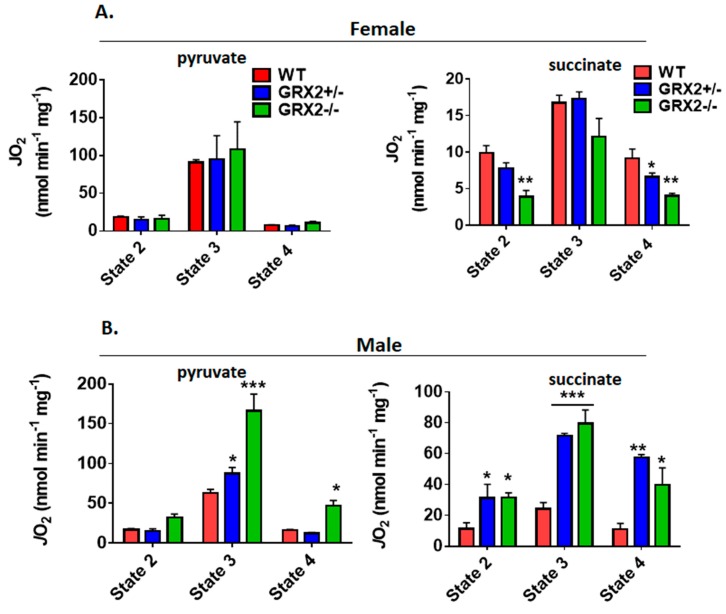
Polarographic measurement of the rate of O_2_ consumption by muscle mitochondria isolated from female and male GRX2-deficient mice and WT littermates. (**A**). Assessment of the three respiratory states of female mitochondria oxidizing pyruvate (10 mM) and malate (2 mM) or succinate (5 mM). (**B**). Assessment of the three respiratory states of male mitochondria oxidizing pyruvate (10 mM) and malate (2 mM) or succinate (5 mM). *n* = 4, mean ± SEM, 1-way ANOVA with a post-hoc Tukey’s test. *: *p* ≤ 0.05, **: *p* ≤ 0.01, ***: *p* ≤ 0.001

It had been found in a previous study that deleting the *Grx2* gene in male mice augments proton-leak-dependent respiration in muscle, an effect that correlated with increased overall energy expenditure and a significant decrease in adipose tissue mass [15]. Additionally, our group recently found that S-glutathionylation of the NDUFS1 subunit in Complex I regulates mitochondrial ATP production and respiration [12]. Measurement of State 2–4 respiration revealed that deleting the *Grx2* had no impact of the bioenergetics of muscle mitochondria isolated from female mice (Figure 6A). Indeed, no differences were observed in ADP-stimulated and proton-leak-dependent respiration in female muscle mitochondria oxidizing pyruvate (Figure 6A). Similar results were collected with mitochondria energized with succinate. Indeed, State 2–4 respiration was similar in succinate-energized mitochondria collected from female WT and GRX2+/− mice (Figure 6A). Intriguingly, however, we found that nonphosphorylating respiration was significantly lower in muscle mitochondria from GRX2−/− female mice (Figure 6A). A trend for decreased ADP-stimulated respiration was also observed but was not significantly different when compared to WT littermates.

Next, we examined the bioenergetics of muscle mitochondria isolated from WT and GRX2-deficient male mice. First, it was observed that WT mitochondria from male mice displayed rates of nonphosphorylating and ADP-stimulated respiration in comparison to female mice when pyruvate served as the substrate (Figure 6B). However, the rate of State 3 respiration increased significantly with the deletion of the *Grx2* gene, with GRX2−/− muscle mitochondria from male mice displaying a rate of O_2_ consumption that was higher than what was observed in samples collected from female mice (Figure 6B). In addition, proton-leak-dependent respiration was also significantly higher in muscle mitochondria collected from GRX2−/− male mice. Similar observations were made with succinate. Indeed, rates of O_2_ consumption in mitochondria from WT male mice were similar to the rates of respiration in female muscle mitochondria. However, deleting the *Grx2* gene induced a significant increase in both nonphosphorylating and ADP-stimulated respiration (Figure 6B).

## 4. Discussion

**Figure 7:**
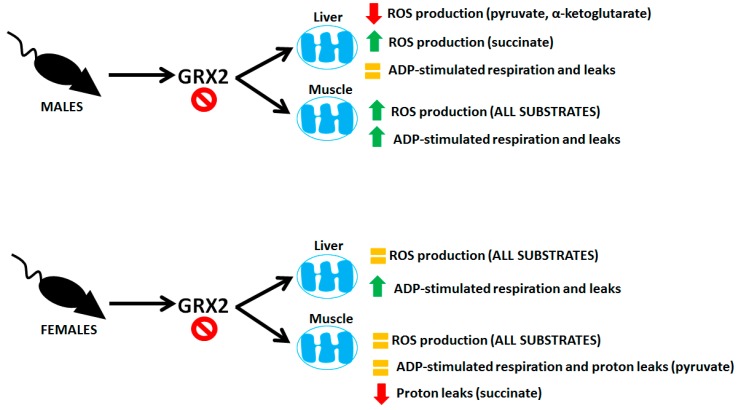
Sex-dependent differences in the bioenergetics of liver and muscle mitochondria isolated from mice containing a deletion for the *Grx2* gene.

Sexual dimorphisms in biochemical and physiological characteristics is a common feature found in animals due to differences in genetic and epigenetic coding and hormonal signaling. Mitochondria display sex-specific differences in function as well, which is related to differences in hormone signaling and expression of mitochondrial proteins encoded in nuclear DNA [22]. Understanding the sexual dimorphisms of mitochondria is critical for health research because dysfunctional bioenergetics can have sex-dependent effects on the manifestation of disorders and metabolic diseases. Protein S-glutathionylation reactions have emerged as an important means of controlling mitochondrial functions. Additionally, deregulation of mitochondrial S-glutathionylation signals can have profound pathological consequences, causing heart disease, hypertension, cataracts, obesity, and perturbations in embryonic development (reviewed in [23]). However, research into the role of these reversible and redox-sensitive switches in modulating bioenergetics has been exclusively carried out using male rodent models [17,24,25,26,27]. Here, we have shown that mice carrying a deletion for the gene encoding GRX2, a thiol oxidoreductase that catalyzes reversible S-glutathionylation of proteins in the matrix, display sex differences in mitochondrial bioenergetics. Our results and the sex differences in the bioenergetics of liver and muscle mitochondria from male and female mice containing the *Grx2* gene deletion are summarized in Figure 7.

Our group had previously shown that deletion of the *Grx2* gene decreases the rate of H_2_O_2_ production by PDH and KGDH in liver mitochondria isolated from male mice [17]. This is associated with the S-glutathionylation of the E2 subunit of PDH and KGDH [17]. Furthermore, the addition of purified GRX2 to liver mitochondria prepared from male mice reverses the S-glutathionylation of PDH and KGDH, restoring the activity and rate of ROS production of both enzyme complexes [6]. In the present study, deleting the *Grx2* gene did not alter ROS production by female liver mitochondria, regardless of the substrate type and concentration. This is in contrast to liver mitochondria from male mice, where deletion of the *Grx2* gene decreased ROS production when pyruvate and α-ketoglutarate served as fuels. This apparent sex-dependent difference could be associated with several factors. First, studies have found that liver, heart, and brain mitochondria from female rodents have better ROS handling characteristics [28,29,30,31]. This is related to higher expression antioxidant defenses, which could account for the lower rate of H_2_O_2_ production in female liver mitochondria when compared to male mice. Indeed, we did observe that WT female mice produced ~3.5-fold and ~5-fold less ROS than their male counterparts when liver mitochondria were energized with pyruvate or α-ketoglutarate. Liver mitochondria from female mice energized with succinate did produce slightly more ROS. However, overall, liver mitochondria from female mice produced less H_2_O_2_ than their male counterparts. Another important feature that may limit ROS production is related to our observation that mitochondria from female rodents display higher rates of ADP-stimulated and nonphosphorylating O_2_ consumption [32]. Liver mitochondria isolated from GRX2-deficient mice displayed significantly higher phosphorylating and proton-leak-dependent O_2_ consumption rates when energized with pyruvate or succinate. The increase in proton leaks could account for the decreased rate of ROS production by decreasing protonic back pressure on the respiratory chain, limiting the number of electrons made available for H_2_O_2_ formation.

Studies aimed at deciphering the impact of S-glutathionylation on the regulation of the bioenergetics of skeletal muscle have also been exclusively carried out with male C57BL/6N mice [7,12,15]. It had been previously demonstrated that deleting the *Grx2* gene in male mice augments respiration and ROS production in muscle mitochondria [15]. It was found that this was related to the deglutathionylation of UCP3, which led to increased proton leaks and fuel combustion [15]. Furthermore, the chemical induction of S-glutathionylation with diamide or disulfiram has the opposite effect—decreasing respiration, leaks, and ROS production in muscle mitochondria, which is related to the modification of Complex I, pyruvate carrier, and UCP3 [7,12]. As mentioned above, mitochondria do carry sex-differences in terms of function, which includes fundamental variances in bioenergetics. For example, muscle mitochondria from female Wistar rats have a higher respiratory capacity, more mitochondrial DNA, and skeletal muscle is able to engage in mitochondrial biogenesis much more easily when compared to males [33]. We made similar observations here, where muscle mitochondria from female WT mice displayed higher rates of ADP-stimulated respiration when pyruvate served as the substrate. Similar observations were also recently made with human muscle where women were found to contain a higher intrinsic capacity for respiration and higher proton leaks [34]. Notably, these bioenergetic characteristics correlate with a higher life expectancy and decreased incidence for development of metabolic diseases in both rats and humans [22]. Based on these previous works, we decided to examine the impact of deleting GRX2 on the bioenergetics of muscle mitochondria prepared from female mice. Several previous studies using male C57BL/6N mice demonstrated that (i) deletion of the *Grx2* gene increases ROS release by muscle mitochondria [15], (ii) elimination of GRX2 augments mitochondrial respiration and proton leaks through UCP3 [15], and (iii) induction of S-glutathionylation lowers respiration and inhibits ROS production [12]. Here, we found no evidence that loss of GRX2 alters the bioenergetics of mitochondria from female mouse muscle. First, it was observed that deleting the *Grx2* gene did not alter ROS production by mitochondria oxidizing pyruvate, α-ketoglutarate, or succinate. Second, loss of GRX2 did not change O_2_ consumption when pyruvate and malate were fueling respiration. In addition, proton-leak-dependent respiration was lower in GRX2-deficient muscle mitochondria oxidizing succinate. This is in contrast to male mice where loss of GRX2 augmented State 3 and 4 respiration in mitochondria energized with pyruvate or succinate, an observation that is consistent with previous studies. Taken together, eliminating the *Grx2* gene has the opposite effect on muscle mitochondria from female mice. These findings suggest that muscle mitochondria from female mice may not be as reliant on using S-glutathionylation and GRX2 to control mitochondrial bioenergetics and ROS production, a finding that is consistent with previous studies showing that important sex dimorphisms exist in muscle fuel metabolism [28].

One surprising observation we made here was that succinate was not a good fuel for supporting respiration in mitochondria isolated from female mice. Indeed, it was found that the respiratory control ratio (RCR) for liver mitochondria isolated from WT male mice was ~5. This is in contrast to liver mitochondria from WT female littermates which displayed significantly lower rates of state 3 respiration (RCR of ~2). Similar observations were made with muscle mitochondria. Pyruvate served as a good fuel for respiration in muscle mitochondria from female mice. However, state 3 respiration was several-fold lower in muscle mitochondria from female mitochondria energized with succinate. A previous study did find that succinate dehydrogenase activity was significantly lower in vastus lateralis from women [35]. In addition, succinate also occurs at a higher concentration in females [36]. These sex differences could account for the lower rate for succinate-supported respiration in mitochondria from female mice observed here. Furthermore, we observed that there was a GRX2-dependent sex difference for succinate-stimulated bioenergetics. Eliminating the *Grx2* gene stimulated succinate-dependent respiration in liver mitochondria from female mice. By contrast, loss of GRX2 had no effect on succinate-supported respiration in liver mitochondria from male mice. Intriguingly, loss of GRX2 had the opposite effect on muscle mitochondria. In male mice, deleting the *Grx2* gene stimulated phosphorylating and proton-leak-dependent respiration, whereas it inhibited nonphosphorylating respiration in female littermates. Taken together, our findings indicate that there is a sex difference in mitochondrial succinate metabolism in liver and muscle tissue.

## 5. Conclusions

Deregulation of protein S-glutathionylation reactions in mitochondria plays a part in metabolic dysfunction, development of heart disease, cataracts, hypertension and obesity, and acute toxicity [13,15,25,26,37,38]. To date, studies relating dysfunctional mitochondrial redox signals to development of diseases has been exclusively carried out with male rodents. Additionally, to our knowledge, only one study so far has shown that there sex differences in cell redox signaling through S-glutathionylation reactions [39]. Due to the strong sex differences that exist in the bioenergetics of mitochondria in mammals and humans, we monitored the rates of H_2_O_2_ production and O_2_ consumption in female mice deficient for GRX2. Importantly, our findings revealed that the partial or full deletion of the *Grx2* gene in female mice does not affect (i) H_2_O_2_ production by liver mitochondria, which was attributed to an increase in ROS handling and respiration and (ii) H_2_O_2_ production or respiration in skeletal muscle mitochondria. Taken together, we have found that deleting the *Grx2* gene revealed some intriguing sex differences in mitochondrial bioenergetics. More work is required to further delineate the impact of deleting GRX2, which drives S-glutathionylation reactions in the matrix, on redox signaling in male and female mitochondria. However, our study suggests that there are potential sex differences associated with the redox control of mitochondrial functions through GRX2-driven S-glutathionylation reactions.
